# Taking appreciation to heart: appreciation at work and cardiovascular risk in male employees

**DOI:** 10.3389/fpubh.2024.1284431

**Published:** 2024-03-04

**Authors:** Alisa Auer, Norbert K. Semmer, Roland von Känel, Livia Thomas, Claudia Zuccarella-Hackl, Roland Wiest, Petra H. Wirtz

**Affiliations:** ^1^Biological Work and Health Psychology, University of Konstanz, Konstanz, Germany; ^2^Centre for the Advanced Study of Collective Behaviour, University of Konstanz, Konstanz, Germany; ^3^Psychology of Work and Organizations, Department of Psychology, University of Bern, Bern, Switzerland; ^4^Department of Consultation-Liaison Psychiatry and Psychosomatic Medicine, University Hospital Zurich, University of Zurich, Zurich, Switzerland; ^5^Department of Clinical Research, University of Bern, Bern, Switzerland; ^6^Institute of Diagnostic and Interventional Neuroradiology, University Hospital Bern,University of Bern, Bern, Switzerland

**Keywords:** appreciation at work, coronary heart disease, blood pressure, HbA1c, blood lipids, coagulation, inflammation, positive cardiovascular health

## Abstract

**Introduction:**

While perceived appreciation at work has been associated with self-reported health and wellbeing, studies considering biological health markers are lacking. In this study, we investigated whether appreciation at work would relate to coronary heart disease (CHD) risk as well as the specificity of this proposed association.

**Methods:**

Our study comprised a total of 103 male participants, including apparently healthy, medication-free, non-smoking men in the normotensive to hypertensive range (*n* = 70) as well as medicated hypertensive and CHD patients (*n* = 33). CHD risk was assessed by blood pressure [mean arterial pressure (MAP)], the diabetes marker glycated hemoglobin A1c (HbA1c), blood lipids [total cholesterol (TC)/high-density lipoprotein-cholesterol (HDL-C) ratio], coagulation activity (D-dimer and fibrinogen), and inflammation [interleukin (IL)-6, tumor necrosis factor-alpha (TNF-α), and C-reactive protein (CRP)]. Perceived appreciation at work, as well as potentially confounding psychological factors (social support, self-esteem, and work strain due to a lack of appreciation), were measured by self-report questionnaires.

**Results:**

We found higher appreciation at work to relate to lower overall composite CHD risk (*p*’s ≤ 0.011) and, in particular, to lower MAP (*p*’s ≤ 0.007) and lower blood lipids (*p*’s ≤ 0.031) in medication-free participants as well as all participants. This overall association was independent of confounding factors, including related psychological factors (*p*’s ≤ 0.049).

**Discussion:**

Our findings indicate that appreciation at work might be an independent health-promoting resource in terms of CHD risk. Implications include that encouraging appreciation at work may help reduce the development and progression of CHD.

## 1 Introduction

“You did a good job!,” “I need your advice, can you help me?,” “Thank you for your assistance!” Statements like these usually induce a wide range of pleasant feelings as a consequence of the implied appreciation (1, 2). In research, appreciation is often captured by similar terms such as respect (3, 4), esteem (5), acknowledgment (6), or recognition (7, 8) that all relate to the same basic idea of valuing someone (9). More precisely, appreciation expresses the recognition of a person’s positive qualities by other people (10, 11). It, for example, signals the value of a person in social relations in terms of positive qualities such as being likable, competent, or moral, and, thus, supports a positive self-image (11, 12).

From a historical perspective, Herzberg et al. (13) were one of the first to propose the positive effects of recognition or appreciation on motivation and job satisfaction. Given its social relevance (12), appreciation is associated with fundamental human motives, including the motive to get along (14) and the need to belong (15). Second, in the context of work stress, appreciation or esteem constitutes one of the three reward factors in the effort-reward imbalance (ERI) model by Siegrist (5). According to that model, work stress results from a perceived imbalance between a person’s efforts in relation to rewards at work, with higher rewards, including esteem, counterbalancing the amount of work stress (5). Notably, esteem, as assessed by the ERI questionnaire, does not quantify the perceived amount of received appreciation but measures the amount of strain that results from the lack of appreciation at work (16). Third, appreciation plays a major role in the stress-as-offense-to-self (SOS) theory (10, 11), in which threats to the self are considered a major source of stress. Here, appreciation is considered a core resource as it boosts self-esteem and may buffer stress experiences resulting from threats to the self. Furthermore, in the context of the SOS theory, a pertinent measure of appreciation has been developed (17). Fourth, in addition to self-esteem, appreciation has also been associated with a further stress-reducing resource, namely social support (18–20). Functional social support includes the two facets of instrumental support and emotional support (19). While instrumental support refers to help concerning the problem at hand, for instance, in terms of tangible help or information, emotional support refers to communicating care, esteem, empathy, and understanding (19, 20). Thus, in the context of social support, appreciation explicitly indicates emotional support (19, 21), but also instrumental support often has an emotional component that transmits appreciation and esteem (20). Nevertheless, there are differences between social support and appreciation. Unlike social support, appreciation can be conveyed in any situation and is often shown in positive situations (e.g., successful cooperation). Not surprisingly, therefore, the effects of appreciation on indicators of wellbeing have been shown to persist after controlling for social support (17, 22, 23). Fifth, recent leadership concepts that focus on health have acknowledged the relevance of recognition or appreciation by supervisors for employee wellbeing (24). Taken together, evidence for the relevance of appreciation, in particular in the context of stress and health, comes from several theories and empirical studies that do not explicitly differentiate appreciation from related constructs, including the reward component of the ERI model (i.e., ERI esteem), social support, and self-esteem. These constructs may intertwine with appreciation and, thus, confound the potential effects of appreciation.

Interestingly, despite the outlined decades of linkage between appreciation and the above-described theories and health-relevant concepts, there are comparatively few studies that quantify perceived appreciation at work as a concept of its own right in the context of wellbeing and health. With respect to *self-reported wellbeing*, there is evidence that higher appreciation at work is associated with higher wellbeing (25), serenity (26), enthusiasm and contentment (22), job satisfaction (4, 9, 17, 27–30), lower feelings of resentment (9, 17), as well as better psychological functioning, represented by a number of psychological resources including self-esteem (31). Regarding *self-reported health*, appreciation has been associated with higher levels of subjective health (30), as well as lower levels of depressive symptoms (22, 32), anxiety (22), emotional exhaustion (33), and lower-back pain (34). In terms of a more *objective assessment of health*, register-based studies show that higher appreciation prospectively predicted a lower risk of sickness absence (35) and fewer early retirements, both based on disability pension (36) as well as self-reported non-disability pension (37). With respect to associations between appreciation and *physiological markers of health*, there is so far only one very recent study that assessed the average situation-specific expectation of appreciation and respect for one’s effort and found higher expectations to relate to lower intima-media thickness (IMT) of the carotid artery (38). Given that the carotid IMT is indicative of the severity of atherosclerosis, the chronic inflammatory process underlying coronary heart disease (CHD) (39–42), the findings of this study point to associations between appreciation and CHD. Notably, this is in line with the concept of positive cardiovascular health (43), where positive psychological wellbeing is proposed to relate to cardiovascular health (44–46). Indeed, concepts related to appreciation, in particular higher social support (47) and lower ERI (48, 49), have been associated with lower CHD risk. However, to the best of our knowledge, data on appreciation and CHD risk are sparse and even absent for a broad measure of appreciation that is not only confined to effort.

In this study, we set out to investigate whether higher perceived appreciation at work would relate to lower CHD risk as assessed by major biological risk factors under resting conditions in male employees with differential CHD risk. To maximize the score ranges and, thus, the variability in CHD risk factors, we included apparently healthy participants in the normotensive to hypertensive range and patients with hypertension or manifest CHD and controlled for relevant confounders. To assess CHD risk in a most comprehensive way, we assessed the major intermediate biological CHD risk factor categories comprising blood lipids, coagulation, and inflammation, in addition to the classical risk factors diabetes and hypertension [e.g., (50–52)]. Apart from diabetes, which was assessed by one marker [glycated hemoglobin A1c, HbA1c, (53)] all other biological risk factors were assessed by at least two major markers to allow for a representative assessment of the respective risk factor category. In detail, as a single-linear measure of hypertension status, mean arterial pressure (MAP) was calculated from systolic (SPB) and diastolic (DBP) blood pressure (BP) (54–56). Moreover, blood lipid assessment included the ratio between total cholesterol (TC) and high-density lipoprotein-cholesterol (HDL-C) (57). Coagulation activity was reflected by D-dimer (58, 59) and fibrinogen (60, 61). Inflammation assessment comprised the acute-phase protein C-reactive protein (CRP) (62, 63) and the cytokines interleukin (IL)-6 and tumor necrosis factor-alpha (TNF-α) (64–66). In addition, we tested the specificity of the proposed association between appreciation and CHD risk by additionally controlling for potential psychological confounders, comprising perceived social support (PSS) and the amount of strain induced by a lack of appreciation (ERI esteem) as conceptually related constructs, and self-esteem as a factor that likely interferes with the perception of appreciation.

## 2 Materials and methods

### 2.1 Study participants

This study is part of a project assessing psychoneurobiological mechanisms in CHD and essential hypertension (67–71). Ethical approval was obtained by the Ethics Committee of the State of Bern, Switzerland, and the study was conducted in accordance with the Declaration of Helsinki principles. All participants provided written informed consent and were financially compensated with 20 Swiss Francs.

For the purpose of the current study, we included hypertensive and normotensive men as well as male patients with diagnosed CHD in order to cover the broadest possible range of cardiovascular risk. Besides the exclusion criteria described below, participants had to be employed at the time of the study assessment, working at least 20 h per week, and have completed the appreciation at work questionnaire in addition to the medical assessment. Due to insufficient medical data, *n* = 6 participants had to be excluded. Furthermore, participants reporting symptoms of infectious disease on the day of the study assessment were excluded (*n* = 3). Our final study sample comprised *N* = 103 participants, with *n* = 25 CHD patients, *n* = 40 hypertensive participants (32 were medication-free and 8 were medicated at study participation), *n* = 32 normotensive participants, and *n* = 6 with white coat or masked hypertension.

#### 2.1.1 Recruitment and general inclusion criteria

The recruitment and assessment of our study participants took place between 2011 and 2016. We invited male CHD patients of the Cardiac Prevention and Rehabilitation Clinic of the Bern University Hospital who had received their diagnosis at least 6 months earlier to participate in our study as described previously (68, 71). Since all CHD patients required CHD medication, we accepted the medication for these patients. Moreover, we recruited apparently healthy (i.e., undiagnosed) medication-free essential hypertensive and normotensive participants with the aid of the Swiss Red Cross of the Canton of Bern as described previously (68, 70). The final classification of essential hypertension and normotension was based on a two-step assessment procedure comprising home and study BP measurements following our previous methods [e.g., (67, 68)] to ensure essential hypertension status validity. Briefly, interested candidates were instructed to measure their BP at home in a seated position after a 15-min rest using sphygmomanometry (Omron M6; Omron Healthcare Europe B.V., Hoofddorp, Netherlands) on three separate days, once in the morning and once in the evening. Based on a maximum of six home BP measurements, participants were preliminarily classified as hypertensive if their average home SBP was ≥ 135 mmHg and/or their average home DBP was ≥ 85 mmHg according to recommendations for home or ambulatory BP measurements (normotensive: SBP < 135 mmHg and DBP < 85 mmHg) (72, 73). To verify the home-measurement-based preliminary classification, trained personnel obtained up to three additional seated baseline BP measurements (see Procedure section). For classification regarding study BP measurements, we applied the standard definition of hypertension based on office or clinic BP measurements and classified participants as hypertensive if their average study SBP was ≥140 mmHg and/or their average study DBP was ≥90 mmHg (normotensive: SBP < 140 mmHg and DBP < 85 mmHg) (72–74). Participants with consistent group assignments based on both home BP and study BP measurements were classified as hypertensive (*n* = 32) or normotensive (*n* = 32). Participants with deviating home and study BP assessments (*n* = 6) were classified as white coat hypertensive if they displayed normotensive home BP and hypertensive study BP (*n* = 3) and as masked hypertensive if they displayed hypertensive home BP and normotensive study BP (*n* = 3). Notably, we accepted diagnosed hypertensive patients and, thus, intake of antihypertensive medication in a small proportion of hypertensive individuals (*n* = 8) to increase sample size, but no other current medication intake (rendering a total of *n* = 40 hypertensive participants).

### 2.2 Procedure

All participants abstained from caffeine and alcohol consumption for 24 h and consumed a semi-standardized breakfast following written instructions prior to arriving at the lab at 8:00 a.m. In addition to the assessment of height and weight, trained personnel obtained up to three additional seated baseline BP measurements on the dominant arm each after a 15-min rest by means of sphygmomanometry (Omron M6; Omron Healthcare Europe B.V., Hoofddorp, Netherlands). To assess CHD risk factors other than BP, blood samples were collected at 11:30 a.m., i.e., after fasting for 3.5 h since arrival.

### 2.3 Psychological measurements

#### 2.3.1 Appreciation at work

Appreciation at work was measured with the 10-item Bern Appreciation Scale, which assesses appreciation by supervisors and co-workers (17). Participants were asked to rate on a 7-point response scale (1 = not at all to 7 = very much) the extent to which statements about different forms of appreciation applied to their work situation, such as compliments, understanding, trust, sympathy, attention, interest, and gratitude [e.g., “My supervisors praise me when I carry out my tasks well” (German: “Mein(e) Vorgesetzte(r) lobt mich, wenn ich meine Aufgaben gut erledige.”); “My colleagues show how much they value my opinion by asking for my advice” (German: “Meine ArbeitskollegInnen fragen mich um Rat und das zeigt mir, dass sie meine Meinung schätzen.”)]. Items were averaged to a total score, with higher scores indicating higher appreciation at work. The psychometric properties of the appreciation total score were found to be adequate, with Cronbach’s α (*N* = 228) = 0.86 (17) and Cronbach’s α (*N* = 103) = 0.89 in our sample.

#### 2.3.2 Social support

PSS was assessed by the 8-item subscale of the Berlin Social Support Scale (BSSS) (75). On a 6-point response scale (1 = strongly disagree to 6 = strongly agree), participants were asked to rate their agreement with statements such as “There are people that offer me help when I need it” (German: “Es gibt Menschen, die mir Hilfe anbieten, wenn ich sie brauche”). Items were averaged to compute the PSS score, with higher scores indicating higher PSS. Psychometric properties are adequate, with Cronbach’s α (*N* = 437) = 0.83 for the PSS subscale (75) and excellent Cronbach’s α (*N* = 103) =0.90 in our sample.

#### 2.3.3 Self-esteem

We used the 10-item German version (76) of the Rosenberg self-esteem scale [RSES; (77)] to measure global self-esteem. Participants are asked to rate positive and negative feelings about themselves [e.g., “On the whole, I am satisfied with myself” (German: “Alles in allem bin ich mit mir selbst zufrieden”)] on a 4-point response scale (1 = strongly disagree to 4 = strongly agree). After recoding where appropriate, items were summarized to a total score, with higher scores indicating higher self-esteem. Psychometric properties have been shown to be adequate with Cronbach’s α (*N* = 4,988) = 0.88 in the reference sample (78) and Cronbach’s α (*N* = 100) = 0.85 in our sample. Notably, *n* = 3 participants (two CHD patients and one normotensive participant) did not fill out the questionnaire.

#### 2.3.4 Strain induced by lack of appreciation (ERI esteem)

To measure the amount of strain that results from the lack of appreciation at work, we used the 5-item esteem subscale of the German version (79) of the ERI questionnaire (16). On a 5-point Likert scale in a two-step rating procedure, participants were asked whether items such as “Considering all my efforts and achievements, I receive the respect and prestige I deserve at work.” (German: “Wenn ich an all die erbrachten Leistungen und Anstrengungen denke, halte ich die erfahrene Anerkennung für angemessen”) would apply or not. If participants indicate a lack of appreciation, they are further asked to rate the resulting extent of distress. Items are summarized to a total score with higher scores indicating lower strain resulting from a lack of appreciation. Psychometric properties have been shown to be adequate (16, 79) with Cronbach’s α (*N* = 666) = 0.76 for the esteem subscale (79) and Cronbach’s α (*N* = 102) =0.73 in our sample. Notably, one normotensive participant did not fill out the questionnaire.

### 2.4 CHD risk assessment

We assessed CHD risk by measuring the following biological risk factors: (1) BP, (2) HbA1c, (3) blood lipid profiles in terms of TC/HDL-C ratio, (4) the prothrombotic factors D-dimer and fibrinogen, and (5) the pro-inflammatory measures IL-6, TNF-α, and CRP. Analyses of HbA1c, blood lipids, and prothrombotic factors were performed in the Center for Laboratory Medicine of the Bern University Hospital (Inselgruppe AG, Bern), while analyses of pro-inflammatory measures were performed in the biochemical laboratory of the Biological Work and Health Psychology group at the University of Konstanz.

#### 2.4.1 HbA1c

For the assessment of the diabetes marker HbA1c, venous blood was drawn into EDTA-coated Monovettes. Analyses were performed with *in vitro* assays for the quantitative determination of HbA1c IFCC (mmol/mol) in whole blood (Tina-quant®, Roche, Mannheim, Germany) using Roche/Hitachi Cobas C Systems (Roche, Mannheim, Germany). The inter- and intra-assay coefficients of variation (CVs) were ≤ 1.6% and ≤ 2.0%, respectively.

#### 2.4.2 Blood lipids

TC and HDL-C were measured from heparin-coated Monovettes (Sarstedt Monovette orange). Analyses were performed using *in vitro* assays (enzymatic colorimetric assays, Roche, Mannheim, Germany) for the quantitative determination of blood lipids in human plasma on a Roche/Hitachi Cobas C Analyzer (Roche, Mannheim, Germany). The inter-and intra-assay CVs were ≤ 1.2% and ≤ 2.5%, respectively.

#### 2.4.3 Coagulation activity

To measure coagulation activity in terms of the prothrombotic factors D-dimer and fibrinogen, venous blood was drawn into polypropylene tubes containing 3.8% sodium citrate (Sarstedt, Nümbrecht, Germany). Citrate tubes were immediately centrifuged for 20 min at 4°C at 2,000 *g*, and plasma was pipetted into aliquots. D-dimer was analyzed using a particle-enhanced immunoturbidimetric assay for the quantitative determination of D-dimers in human plasma (INNOVANCE® D-Dimer, Siemens Healthcare GmbH, Erlangen, Germany) on a Sysmex CS-5100 (Sysmex Europe, Norderstedt, Germany). Plasma fibrinogen levels were determined by a routine clotting assay applying standard quality procedures following the Clauss method. The intra- and inter-assay CVs were ≤ 7.9%.

#### 2.4.4 Pro-inflammatory measures

For the assessment of inflammation in terms of the pro-inflammatory measures IL-6, TNF-α, and CRP, venous blood was drawn in EDTA-coated Monovettes (Sarstedt, Nümbrecht, Germany) and immediately centrifuged for 10 min at 2,000 *g* and 4°C. The obtained plasma was stored at −80°C until analysis. The IL-6 and TNF-α levels were determined with a high-sensitivity chemiluminescence sandwich immunoassay (Meso Scale Discovery (MSD), Rockville, USA). CRP was determined using a high-sensitivity enzyme immunoassay (ELISA, IBL Hamburg, Germany). For IL-6, inter- and intra-assay CVs were ≤ 7.3% and ≤ 4.5%. For TNF-α, inter- and intra-assay CVs were ≤ 10.1% and ≤ 3.4%. For CRP, inter- and intra-assay CVs were ≤ 6.3 % and ≤ 6.9%. Notably, CRP could not be analyzed in 14 participants due to problems with blood sampling or processing or an insufficient amount of samples for the analysis.

### 2.5 Statistical analyses

Data were analyzed using SPSS (Version 29.0) packages for Macintosh (IBM SPSS Statistics, Chicago, IL, USA) and presented as mean ± standard error of the mean (SEM). All tests were two-tailed, with the significance level set at *p* < 0.05.

We *a-priori* calculated the statistical power analyses using the statistical software G∗Power for Macintosh (Version 3.1.9.6; Heinrich Heine University Düsseldorf, Germany). To conservatively allow for the detection of small to medium effect sizes of *R*^2^ = 0.10 in linear regression analyses, the required sample size to obtain a power of (1 – *β*) = 0.80 is *N* = 74.

For all participants, we calculated MAP based on up to three study BP measurements by the formula MAP = (2/3*mean study DBP) + (1/3*mean study SBP), as well as body mass index (BMI) by the formula BMI = kg/m^2^. To compute an aggregated coagulation index, we averaged z-transformed levels of D-dimer and fibrinogen. For an aggregated inflammatory index, we accordingly averaged z-transformed levels of IL-6, TNF-α, and CRP. Notably, as CRP could not be analyzed in 14 participants, the aggregated inflammatory index for these participants consists of averaged z-transformed levels of IL-6 and TNF-α.

All data were tested for normal distribution using the Kolmogorov–Smirnov test. We used non-normality robust multivariate analyses of covariance (MANCOVAs) (80) and conducted statistical analyses with original data. Effect size parameters partial η^2^ [η^2^_p_; effect size conventions η^2^_p_: 0.01 = small; 0.06 = medium; 0.14 = large (81)] and *R*^2^ changes [Δ*R^2^*; effect size conventions *R*^2^: 0.02 = small; 0.13 = medium; 0.26 = large (81)] are reported where appropriate.

To test whether appreciation at work is associated with cardiovascular risk, we calculated MANCOVAs. Thereby, appreciation at work was included as a linear independent variable, and MAP, HbA1c, TC/HDL-C ratio, the coagulation index, and the inflammatory index were entered as dependent variables, yielding the latent variable CHD risk. MANCOVAs were conducted without and with control for possible confounding effects of medication intake (antihypertensive medication, blood lipid and/or diabetes medication, anticoagulation medication, and other medication intake), age, BMI, and smoking (51, 82). Moreover, to completely exclude potential confounding effects of medication intake or smoking, we repeated our analyses with medication-free, non-smoking participants (*n* = 70) without and with controlling for age and BMI. *Post-hoc* testing of significant multivariate effects of appreciation at work comprised hierarchical linear regression analyses with each dependent variable separately regressed on appreciation at work in order to identify the direction of the effect and the variables that mainly account for it.

To test whether appreciation at work would buffer cardiovascular risk independent of important related psychological factors, we repeated the above-described procedure while additionally considering PSS, self-esteem, and/or ERI esteem as covariates.

## 3 Results

### 3.1 Group characteristics

Table 1 provides group characteristics of the 103 male participants, including the 70 medication-free, non-smoking participants. Our study sample comprised employees from various organizations pursuing a wide variety of jobs, such as agriculturists, police officers, computer scientists, consultants, or project managers. Our participants worked an average of 43.35 h/weeks (SEM = 0.68; range = 20–58). The mean age was 50.15 years (SEM = 0.98; range = 25–71) and the mean BMI was 27.46 kg/m^2^ (SEM = 0.36; range = 19.78–38.90).

**Table 1:** Group characteristics.

	All participants *N* = 103	Medication-free, non-smoking participants *n* = 70
Group assignment	*n* = 25 CHD patients *n* = 40 HT *n* = 32 NT *n* = 6 wc/m HT	*n* = 32 HT *n* = 32 NT *n* = 6 wc/m HT
Age (years)	50.15 ± 0.98 (25–71)	48.29 ± 1.23 (25–71)
BMI (kg/m^2^)	27.46 ± 0.36 (19.78–38.90)	27.27 ± 0.46 (19.78–38.86)
Smoking [% (*N*)]	3.88% (*n* = 4)	-
Working hours	*n* = 100 43.35 ± 0.68 (20–58)	*n* = 69 43.40 ± 0.76 (20–55)
Appreciation at work	5.37 ± 0.09 (3.10–6.90)	5.38 ± 0.10 (3.10–6.90)
Perceived social support	5.18 ± 0.06 (3.50–6.00)	5.19 ± 0.08 (3.50–6.00)
Self-esteem	*n* = 100 34.35 ± 0.43 (20–40)	*n* = 69 34.32 ± 0.44 (25–40)
Amount of stress induced by lack of appreciation	*n* = 102 23.29 ± 0.28 (11–25)	*n* = 69 23.42 ± 0.35 (11–25)
BP (study)		
SBP (mmHg)	138.23 ± 1.54 (109.33–189.67)	138.43 ± 1.92 (109.33–189.67)
DBP (mmHg)	85.03 ± 1.12 (58.33–115.00)	86.00 ± 1.42 (58.33–115.00)
MAP (mmHg)	102.76 ± 1.22 (75.33–139.89)	103.47 ± 1.54 (75.33–139.89)
HbA1c (nmol/mol)	37.08 ± 0.39 (28–50)	36.27 ± 0.45 (28–49)
TC/HDL-C ratio	3.76 ± 0.09 (2.01–6.41)	3.96 ± 0.12 (2.01–6.41)
Coagulation		
Fibrinogen (g/L)	2.58 ± 0.05 (1.47–3.97)	2.55 ± 0.06 (1.47–3.97)
D-Dimer (μg/L)	441.28 ± 26.45 (45–1,481)	458.50 ± 34.59 (45–1,481)
Inflammation		
IL-6 (pg/mL)	0.51 ± 0.03 (0.16–1.52)	0.48 ± 0.03 (0.16–1.52)
TNF-α (pg/mL)	1.98 ± 0.06 (0.70–4.91)	1.95 ± 0.08 (0.70–4.91)
CRP (μg/mL)	*n* = 89 2.43 ± 0.22 (0.07–9.59)	*n* = 58 2.66 ± 0.29 (0.11–9.59)

Values are means ± SEM (range) if not indicated differently; BMI, body mass index; BP, blood pressure; SBP, systolic blood pressure; DBP, diastolic blood pressure; MAP, mean arterial pressure; TC/HDL-C ratio, total cholesterol/high-density lipoprotein-cholesterol ratio; IL-6, interleukin-6; TNF-α, tumor necrosis factor-alpha; CRP, Creactive protein; CHD patients, patients with coronary heart disease; NT, normotensive participants; HT, hypertensive participants; wc/m HT, white coat or masked hypertension; *n* = sample size; deviating sample sizes of a parameter are indicated.

### 3.2 Appreciation at work and cardiovascular risk

**Figure 1:**
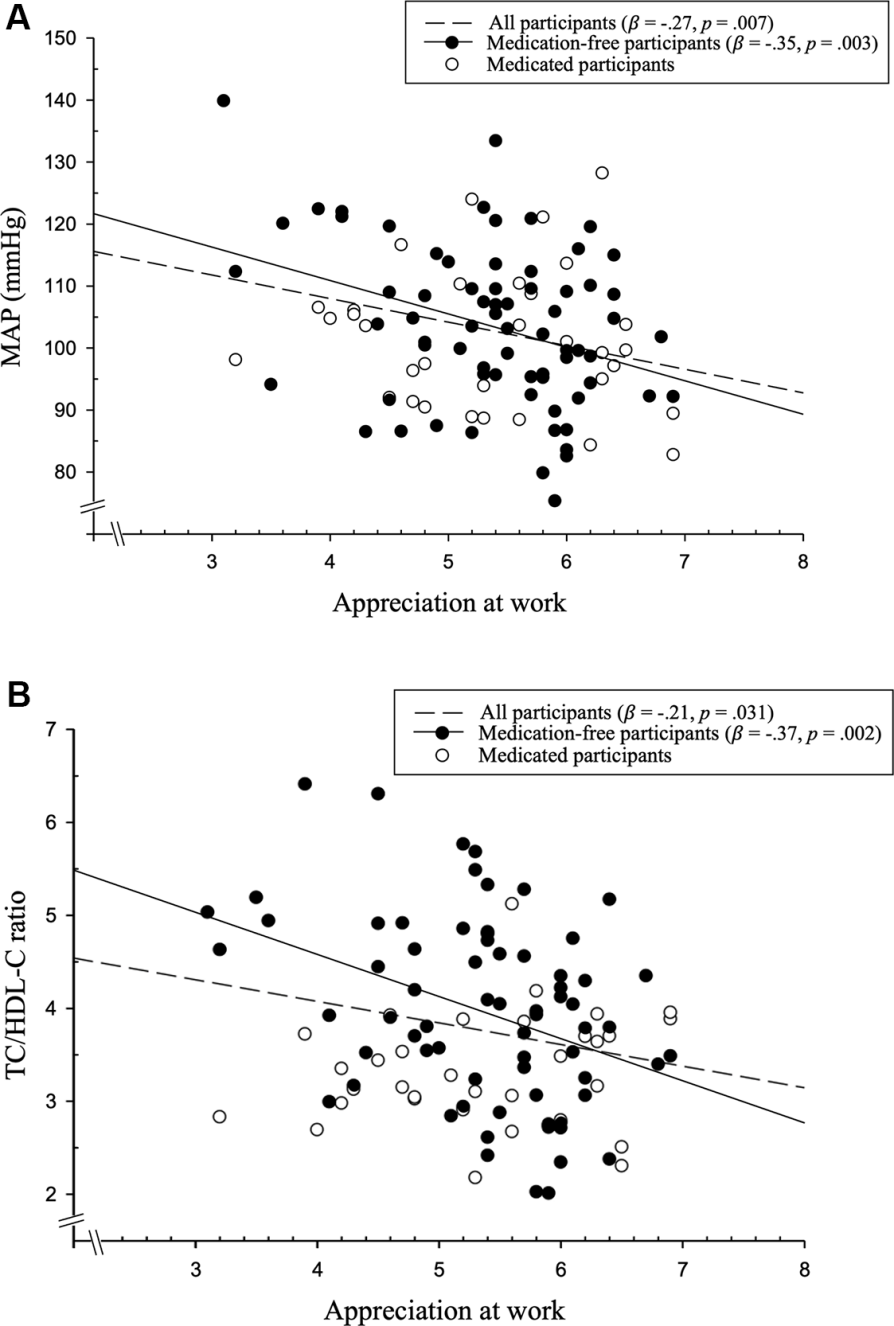
Higher appreciation at work significantly related to **(A)** lower mean arterial pressure (MAP) (*β* = −0.27, *p* = 0.007, *R^2^* = 0.07) and **(B)** lower blood lipids (total cholesterol (TC)/high-density lipoprotein-cholesterol (HDL-C) ratio) (*β* = −0.21, *p* = 0.031, *R^2^* = 0.05).

Over all participants (*N* = 103) MANCOVAs revealed that appreciation at work was significantly related to CHD risk in terms of the dependent variables MAP, HbA1c, TC/HDL-C ratio, the coagulation index, and the inflammatory index. Associations were significant both without (*p* = 0.011, η^2^_p_ = 0.14) and with controlling for medication intake, age, BMI, and smoking (*p* = 0.046, η^2^_p_ = 0.12). *Post-hoc* hierarchical linear regression analyses indicated that higher appreciation at work was associated with lower MAP (*β* = −0.27, *p* = 0.007, see Figure 1A; with control variables: *β* = −0.19, *p* = 0.026) and lower blood lipids (TC/HDL-C ratio) (*β* = −0.21, *p* = 0.031, see Figure 1B; although with control variables, there was only a trend: *β* = −0.17, *p* = 0.079). There were no associations either with HbA1c or the coagulation and inflammatory indices (*p*’s ≥ 0.11). Multivariate effects became stronger when repeating MANCOVAs in medication-free, non-smoking participants (*n* = 70) (*p* = 0.008, η^2^_p_ = 0.21; with control variables age and BMI: *p* = 0.018, η^2^_p_ = 0.19). Again, higher appreciation at work was associated with lower MAP (*β* = −0.35, *p* = 0.003; with control variables: *β* = −0.25, *p* = 0.011) and lower blood lipids (TC/HDL-C ratio) (*β* = −0.37, *p* = 0.002; with control variables: *β* = −0.32, *p* = 0.005), but not with HbA1c, or the coagulation and inflammatory indices (*p*’s ≥ 0.15). The results are depicted in more detail in Table 2.

**Table 2:** Results of MANCOVAs (with appreciation at work predicting the latent variable CHD risk with MAP, HbA1c, TC/HDL-C ratio, the coagulation index, and the inflammatory index as dependent variables) in all participants as well as in medication-free, non-smoking participants, and post-hoc linear regression analyses with each dependent variable separately regressed on appreciation at work.

	All participants (*N* = 103) Without covariates (with covariates of medication intake, age, BMI, and smoking)					Medication-free, non-smoking participants (*n* = 70) Without covariates (with covariates of age andBMI)				
MANCOVAs	[*df_Num_, df_Den_*]	*F*	*p*	η^2^_p_	*Wilk’s* Λ	[*df_Num_, df_Den_*]	*F*	*p*	η^2^_p_	*Wilk’s* Λ
Appreciation at work	[5, 97] (5, 90)	3.16 (2.36)	**0.011 (0.046)**	0.14 (0.12)	0.86 (0.88)	[5, 64] (5, 62)	3.49 (2.98)	**0.008 (0.018)**	0.21 (0.19)	0.79 (0.81)
*Post-hoc* linear regression analyses		*β*	*p*	(Δ)*R^2^*			*β*	*p*	(Δ)*R^2^*	
MAP		**−0.27 (−0.19)**	**0.007 (0.026)**	0.07 (0.04)			**−0.35 (−0.25)**	**0.003 (0.011)**	0.12 (0.06)	
HbA1c		−0.14 (−0.12)	0.15 (0.24)				0.05 (0.06)	0.70 (0.66)		
TC/HDL-C ratio		**−0.21** (−0.17)	**0.031** (0.079)	0.05 (0.03)			**−0.37 (−0.32)**	**0.002 (0.005)**	0.14 (0.10)	
Coagulation index		0.15 (0.16)	0.14 (0.11)				0.17 (0.18)	0.15 (0.15)		
Inflammation index		−0.10 (−0.03)	0.34 (0.72)				−0.04 (0.01)	0.77 (0.95)		

MAP, mean arterial pressure; TC/HDL-C ratio, total cholesterol/high-density lipoprotein-cholesterol ratio; coagulation index = averaged z-transformed levels of D-dimer and fibrinogen; inflammation index = averaged z-transformed levels of interleukin-6 (IL-6), tumor necrosis factor-alpha (TNF-α), and C-reactive protein (CRP), *n* = sample size; df_Num_ = degrees of freedom numerator; df_Den_ = degrees of freedom denominator; significant values are highlighted in bold (*p* < 0.05).

To test for specificity, i.e., whether appreciation at work is associated with CHD risk independent of other related psychological constructs, we repeated the above-described analyses and tested whether additional control for PSS, self-esteem, and the amount of strain that results from the lack of appreciation at work (ERI esteem) as covariates either alone or combined would change the obtained results. The results showed that appreciation at work was independently related to CHD risk (*p*’s ≤ 0.042, η^2^_p_’s ≥ 0.12; with control variables: *p*’s ≤ 0.049, η^2^_p_’s ≥ 0.12). Neither PSS, self-esteem, nor ERI esteem were associated with CHD risk independent of appreciation (*p*’s ≥ 0.14; with covariates: *p*’s ≥ 0.11). These results were obtained in analyses including all participants as well as medication-free, non-smoking participants only.

## 4 Discussion

In this study, we investigated whether appreciation at work would relate to CHD risk as assessed by major biological risk factors under resting conditions, i.e., MAP, HbA1c, TC/HDL-C ratio, coagulation, and inflammation. We further tested for the specificity of the proposed association by additionally controlling for important related psychological factors, i.e., PSS, self-esteem, and the amount of strain induced by a lack of appreciation.

We found that higher appreciation at work was significantly and independently associated with lower overall CHD risk and, in particular, with lower MAP and lower blood lipid levels. These findings corroborate and extend studies that found appreciation at work to be associated with self-reported health and wellbeing (4, 9, 17, 22, 25–37) to health assessment in terms of biological CHD risk factors. The observed association between perceived appreciation at work and biological CHD risk is in line with the study that found the appreciation for one’s effort to relate to lower IMT (38). Moreover, the specificity of our results, i.e., that appreciation at work relates to CHD risk independent of PSS, self-esteem, or the amount of strain that results from the lack of appreciation, indicates that the amount of perceived appreciation at work seems to be an independent driving resource with regard to CHD risk.

What *mechanisms* may underlie the observed association between higher appreciation at work and lower CHD risk? First, on the basis of the existing theory, we propose that appreciation may buffer stress effects. Given that work stress is a risk factor for CHD (48, 83, 84) and appreciation is considered a major resource in the context of work stress according to the ERI model (5) and the SOS theory (10, 11), we consider appreciation to counteract and, thus, prevent adverse health consequences of work stress. This reasoning is in line with the job-demand resources model (JD-R), with job resources postulated to buffer the negative effects of job demands on strain and health (85, 86). Indeed, appreciation at work has been shown to buffer the negative effects of illegitimate tasks (22), long working hours (17), or work interruptions (23) on indicators of health and wellbeing. Second, we propose positive effects of appreciation on cardiovascular health, independent of stress. Appreciation induces a wide range of positive feelings (1, 2, 22, 26, 87) and positive psychological wellbeing, including positive affect, has been shown to relate to lower CHD risk, both in cross-sectional and prospective studies (44, 88). Indeed, based on the concept of positive cardiovascular health (43), biological (e.g., lower BP, lower lipids, and lower inflammation), behavioral (e.g., smoking cessation, healthy diet, and physical activity), and psychosocial pathways have been proposed to underlie the association between positive psychological wellbeing and better cardiovascular health (45, 46, 89). Given this, we assume that the observed association between appreciation and better cardiovascular health may similarly be mediated by positive affective pathways. However, this remains to be studied. Taken together, appreciation may not only buffer stress effects with the associated increase in cardiovascular risk but also actively contribute to better cardiovascular health. Given the specificity of our results, future research is needed to further support the evident role of appreciation in cardiovascular health. Moreover, the potential underlying mechanisms remain to be elucidated.

The *implications* of our findings include encouraging appreciation at work and investigating whether such interventions will ultimately improve cardiovascular health in employees in terms of reducing cardiovascular risk factors and a better prognosis. In general, there are different interaction levels to express appreciation. These interaction levels include the organizational (e.g., policies and programs stating the organization’s intention to recognize the work performed by its members), vertical (top-down and bottom-up), horizontal (between team members), external (e.g., in service occupations customers), and social (community’s appreciation of the organization or its social value) level (90). Moreover, different forms of appreciation include personal appreciation and an appreciation of results, work practice, or job dedication (90), but also the assignment of interesting tasks and job design in general (11). For example, in their diary study, Stocker et al. (26) found that appreciation in small-scale, simple, and economic ways, such as praise and gratitude, predicted positive effects at the end of a workday. However, there are indications that a discrepancy between employees’ expectations of appreciation and perceived appreciation at work, especially with regard to supervisors, is not uncommon (6, 91). Reasons for this discrepancy may include the underestimation of the positive value and the overestimation of the awkwardness of explicitly expressing appreciation, which may prevent expressing appreciation (92). To allow for an organizational culture that encourages the expression of appreciation, organizations should actively raise awareness of the positive effects of appreciation as well as ways to provide authentic appreciation in order to counteract the potential overestimation of awkwardness. According to Yukl (8), one should pay attention to (1) recognizing a variety of contributions and achievements, (2) actively searching for contributions to recognize, (3) recognizing improvements in performance, (4) recognizing commendable efforts even if they failed, (5) not limiting recognition to high-visibility jobs, (6) not limiting recognition to a few best performers, (7) providing specific recognition, (8) providing timely recognition, and (9) using an appropriate form of recognition. Finally, expressing appreciation might not only have a positive impact on employees’ health and wellbeing but also have further positive consequences. For instance, on the organizational level, appreciation has been associated with higher work engagement (7, 93, 94), lower turnover intentions (95, 96), higher intrinsic motivation (97–99), and task performance (100). Moreover, positive cross-over effects into the family domain have been found (30, 93).

The *strengths* of our study include the assessment of CHD risk by major independent biological risk factors under resting conditions. In addition, to maximize the score ranges in CHD risk factors, we included apparently healthy participants in the normotensive to hypertensive range and medicated hypertensive and CHD patients. Moreover, we controlled for a variety of potential confounding variables and tested for the specificity of the proposed association. The *limitations* of our study comprise the limited generalizability of our results beyond middle-aged, employed men. Furthermore, we recruited blood donors, which, however, should not further limit the generalizability of our findings (101). Notably, given that we included employees from various organizations pursuing a wide variety of jobs, our results are unlikely to be driven by a specific organization or occupation. Nevertheless, potential occupation-specific differences as well as the effects of working conditions, including shift work, should be scrutinized in future studies. Moreover, as the meaning and importance of appreciation may diverge in different cultures, further studies in other cultures are needed (102). In addition, the medication of hypertensive and CHD patients could have confounded their CHD risk. However, analyses including all participants as well as medication-free, non-smoking participants yielded very similar results. Similarly, white coat or masked hypertension may confound CHD risk. However, complementary analyses after excluding white coat or masked hypertensive participants (*n* = 6) did not significantly change results (data not shown). In addition, we did not control for physical (in)activity, another important classical CHD risk factor (103, 104). Notably, future studies should assess and consider health behaviors, such as physical activity, given that health behaviors could constitute a potential pathway underlying the association between appreciation at work and CHD risk (45, 46, 89, 105). Moreover, our results are cross-sectional and do not allow causal conclusions, and the possible impact of third variables cannot be ruled out. For example, participants who were especially healthy might have triggered more appreciation due to superior performance and positive social behavior.

Taken together, we found higher appreciation at work to significantly and independently relate to lower overall CHD risk, and, in particular, to lower MAP and lower blood lipids. In other words, the current study provides further evidence indicating that appreciation at work is an important resource for health and wellbeing. Future studies are needed to verify the observed results and to elucidate whether our findings also apply to women and across cultures. Moreover, it should be investigated if different interaction levels, forms, or sources of appreciation may differ in their associations with health and wellbeing. In addition, future research is needed to determine causality and elucidate underlying mechanisms.

## Data availability statement

The raw data supporting the conclusions of this article will be made available by the corresponding author, without undue reservation.

## Ethics statement

The study involving human participants was approved by Ethics Committee of the State of Bern, Switzerland [Kantonale Ethikkomission Bern (KEK), reference number KEK-BE: 215/10]. The study was conducted in accordance with the local legislation and institutional requirements. The participants provided their written informed consent to participate in this study.

## Author contributions

AA: Data curation, Formal analysis, Visualization, Writing – original draft. NKS: Resources, Writing – review & editing. RvK: Funding acquisition, Resources, Supervision, Writing – review & editing. LT: Data curation, Investigation, Writing – review & editing. CZ-H: Data curation, Investigation, Writing – review & editing. RW: Writing – review & editing. PHW: Conceptualization, Formal analysis, Funding acquisition, Supervision, Visualization, Writing – original draft.
